# Editorial: Understanding pathogen spread in invasive vertebrate species

**DOI:** 10.3389/fvets.2026.1869931

**Published:** 2026-05-15

**Authors:** Felipe A. Hernández, André V. Rubio, Andrés M. López-Pérez

**Affiliations:** 1 Instituto de Medicina Preventiva Veterinaria, Facultad de Ciencias Veterinarias, Universidad Austral de Chile, Valdivia, Chile; 2 Departamento de Ciencias Biológicas Animales, Facultad de Ciencias Veterinarias y Pecuarias, Universidad de Chile, Santiago, Chile; 3 Red de Biología y Conservación de Vertebrados, Instituto de Ecología A.C. Xalapa, Veracruz, Mexico

**Keywords:** disease ecology, invasive species, One Health, pathogen spread, wildlife-livestock interface, zoonosis

The global rise of biological invasions, particularly by vertebrate species, represents one of the most pressing challenges for biodiversity conservation, ecosystem functioning, and public health (1, 2). Invasive vertebrates can act as efficient disseminators of pathogens, a phenomenon increasingly recognized as “pathogen pollution” (3). Through their high adaptability, mobility, and often high population densities, invasive species can facilitate the introduction and spillover of multi-host pathogens into naïve ecosystems, affecting native wildlife, domestic animals, and humans (4, 5).

Within this context, the present Research Topic brings together four original studies that explore different facets of pathogen spread in invasive vertebrate species across diverse geographical and ecological settings. Collectively, these contributions highlight the epidemiological significance of invasive taxa and underscore the need for integrated surveillance under a One Health framework.

One of the most insidious pathways of pathogen dissemination is through invasive commensal rodents. Calvo-Mac et al. investigated the occurrence of *Leptospira* spp., *Toxoplasma gondii*, and respiratory viruses in invasive black rats (*Rattus rattus*) inside and outside a coastal protected area in southern Peru. They found a 34% molecular detection frequency of pathogenic *Leptospira* in rat blood, with significantly higher occurrence in human-impacted zones outside the reserve (54.2%) compared to inside (17.2%). Notably, the study detected *T. gondii* antibodies in one rat, but no evidence of avian influenza or other respiratory viruses. These findings suggest that anthropogenic environments may amplify leptospiral circulation, and that invasive rats can act as sentinels of zoonotic risk at the wildlife-domestic-human interface (6, 7). Furthermore, the absence of respiratory viruses in this study adds to the growing scrutiny on whether rodents can maintain highly pathogenic avian influenza viruses (8, 9).

In a complementary study focusing on the same invasive rodent, Alvarez Rojas et al. examined the genetic diversity and infection patterns of the cestode *Hydatigera taeniaeformis* in black rats from the temperate rainforest of southern Chile. Using mitochondrial *cox1* barcoding, they identified only two haplotypes, both closely related to sequences from Peru, Africa, Europe, and Australia, supporting the hypothesis of a recent introduction linked to Spanish colonization (10). Interestingly, the same haplotypes were found in road-killed kodkods (*Leopardus guigna*), confirming the role of wild felids as definitive hosts of *H. taeniaeformis* in southern South America (11). The prevalence of *H. taeniaeformis* in rats was 19.2%, and parasite length correlated positively with host body size and weight. These results illustrate how invasive rodents can participate in the life cycle of parasites that involve both invasive and native definitive hosts, with potential implications for the health of vulnerable wild carnivores (12).

Beyond rodents, other invasive vertebrates can also act as bridge hosts for pathogens of concern. Mariacher et al. documented the first detection of low pathogenic avian influenza virus (LPAIV) subtype H5N2 in an African sacred ibis (*Threskiornis aethiopicus*) culled in Tuscany, Italy. The virus carried two mammalian adaptation markers (S155N and T156A) in the HA protein, although the overall zoonotic risk was considered low. Phylogenetic analysis showed that the virus clustered with European LPAI strains, while the PA gene segment grouped with Asian-Russian isolates from 2021 to 2022. The sacred ibis is known to forage in wetlands and landfills, often in proximity to poultry farms, raising concerns about its potential as a bridge host for avian influenza transmission between wild reservoirs and domestic birds (13, 14). This study emphasizes that invasive birds should not be overlooked in avian influenza surveillance programs.

Finally, the Enemy Release Hypothesis (ERH) was explored by Suwannarat et al. in the blackchin tilapia (*Sarotherodon melanotheron*), a cichlid fish recently introduced to Thailand. Across 164 individuals collected from diverse environments (sea, estuary, canal, shrimp farms), no helminths were detected, contrasting with the high parasite diversity reported in its native African range (15, 16). The absence of helminths was attributed to the recent introduction (4–8 years), which may not have allowed sufficient time for parasite accumulation, consistent with the ERH (17, 18). This parasite release could confer a competitive advantage to the tilapia, facilitating its rapid spread in Thai waters. Interestingly, the study did not find zoonotic trematodes, which are common in Southeast Asian aquaculture, suggesting that the blackchin tilapia may not currently pose a significant food-safety risk, although continued monitoring is warranted (19).

Taken together, these four studies provide compelling evidence that invasive vertebrates—from rodents and fish to birds—can play diverse roles in pathogen dynamics, acting as reservoirs, sentinels, and bridge hosts. They highlight the importance of integrating molecular diagnostics, ecological context, and historical biogeography to understand and predict pathogen spread. As invasive species continue to expand their ranges under global change, proactive surveillance and control measures are essential to mitigate risks to wildlife, domestic animals, and human health, fully embracing the One Health paradigm (20, 21).
